# 

**Published:** 1872-07

**Authors:** 


### ATLANTA MEDICAL AND SURGICAL JOURNAL.

EDITED BY

JOSEPH P. LOGAN, M.D.,

AND

W. F. WESTMORELAND, M.D.,

*Professor of the Principles and Practice of Surgery in Atlanta Medical College.*

S.G.O. INDEXED

VOL. X.—JULY, 1872.—No. 4.

*PAX ET SCIENTIA, SED VERITAS SINE TIMORE.*

ATLANTA, GEORGIA:

PLANTATION PUBLISHING COMPANY'S PRESS.

1872.

